# Genome-based reclassification of the genus Lactococcus and two novel species Pseudolactococcus yaeyamensis gen. nov., sp. nov. and Lactovum odontotermitis sp. nov. isolated from the gut of termites

**DOI:** 10.1099/ijsem.0.006803

**Published:** 2025-06-04

**Authors:** Kota Abe, Masahiro Yuki, Yumiko Imagawa, Atsushi Hisatomi, Moriya Ohkuma, Mitsuo Sakamoto, Satoko Noda

**Affiliations:** 1 Graduate School of Science and Engineering, Ibaraki University, Mito, Ibaraki 310-8512, Japan; 2 Microbe Division/Japan Collection of Microorganisms, RIKEN BioResource Research Center, Tsukuba, Ibaraki 305-0074, Japan; 3 NODAI Culture Collection Center, Tokyo NODAI Research Institute, Tokyo University of Agriculture, Setagaya-ku, Tokyo 156-8502, Japan

**Keywords:** *Lactococcus*, *Lactovum*, *Streptococcaceae*, termite

## Abstract

The genus *Lactococcus* was proposed by Schleifer *et al*. by separating *Lactococcus lactis* from the genus *Streptococcus*. Although the family *Streptococcaceae* consists of four genera, each genus contains a relatively small number of species, with the exception of the genus *Streptococcus*, which contains more than 100 species. The genera *Lactococcus* and *Lactovum* currently comprise 26 species and a single species, respectively. This study evaluated the taxonomy of the genus *Lactococcus* based on the 16S rRNA gene phylogeny, core-genome phylogeny and (conserved) pairwise average amino acid identity. These evaluations clearly indicated that the genus *Lactococcus* could be divided into two genus-level clusters, and we propose to reclassify this genus into two; the authentic *Lactococcus*, which includes the *L. lactis* group, and a novel genus for which the name *Pseudolactococcus* is proposed. Three lactic acid bacterial strains, RyT2^T^, OfM1^T^ and OfM2, were isolated from the gut of termites in Okinawa, Japan. Based on the combination of genetic and phenotypic data, we conclude that these isolates represent two novel species of the genera *Pseudolactococcus* and *Lactovum*, respectively, for which we propose the names *Pseudolactococcus yaeyamensis* sp. nov. (RyT2^T^=JCM 36015^T^=DSM 118067^T^) and *Lactovum odontotermitis* sp. nov. (OfM1^T^=JCM 34431^T^=DSM 118066^T^, OfM2=JCM 34432), respectively.

## Introduction

The genera *Lactococcus* and *Lactovum* belong to the family *Streptococcaceae* and are typical lactic acid fermenters. The family *Streptococcaceae* currently consists of four genera: *Floricoccus*, *Lactococcus*, *Lactovum* and *Strepotococcus*. In this family, each genus contains a relatively small number of species, with the exception of the genus *Streptococcus*, which contains more than 100 species.

Lactic acid bacteria are found in many habitats, including dairy products, milk, vegetables and activated sludge [1,3]. Members of the genus *Lactococcus* have a diverse host range, including insects, and have traditionally been used in the production of many important fermented foods. In fact, several species have recently been found in association with animals other than cattle, such as fish, sugar gliders and termites [4,11]. Twenty-six species have been described in the genus *Lactococcus*, and previous phylogenetic studies, including recently described species, have shown that the genus *Lactococcus* has two clades. *Lactovum miscens* has been isolated from an acidic soil environment and comprises a single species in this genus [12].

The symbiotic association of micro-organisms has a profound effect on host adaptation to the environment, and this association contributes to niche expansion, particularly for insect hosts [13,15]. These symbiotic associations were mostly conserved between host species, even when collected from geologically distant habitats [16,17]. Recently, several lactic acid bacteria such as *Lactococcus* species have been isolated from the gut of termites and described as new species [4,5, 9, 10]. In this study, based on a phylogenetic study, we propose the reclassification of the genus *Lactococcus* into two genera. One is the authentic *Lactococcus*, which includes the *Lactococcus lactis* group, and the other is a novel genus for which the name *Pseudolactococcus* is proposed. In addition, we characterized strains derived from two termite species, designated RyT2^T^, isolated from the gut of *Reticulitermes yaeyamanus*, and two strains, OfM1^T^ and OfM2, isolated from the gut of *Odontotermes formosanus*, and proposed that these strains represent two novel species of the genera *Pseudolactococcus* and *Lactovum*, respectively.

## Isolation and ecology

Host termite colonies of *R. yaeyamanus* were collected from Iriomote Island and those of *O. formosanus* from Main Island in Okinawa, Japan. The termite colonies were maintained in plastic boxes at room temperature before use. After washing the surface of the body of worker termites with sterile water, their gut was removed from the body with sterile forceps and squeezed in sterile 0.4% (w/v) NaCl solution. The gut contents of *R. yaeyamanus* and *O. formosanus* were spread on the trypticase soy (TS) plates and de Man–Rogosa–Sharpe (MRS) plates, respectively. Each plate was cultivated at 30 °C for 2 days under anaerobic conditions using the AnaeroPack system (Mitsubishi Gas Chemicals, Tokyo, Japan). The bacterial strain RyT2^T^ was isolated from *R. yaeyamanus*. Two bacterial strains, OfM1^T^ and OfM2, were isolated from *O. formosanus*. These strains were preserved by lyophilization and freezing at –80 °C. Strains RyT2^T^, OfM1^T^ and OfM2 were deposited in the JCM and DSMZ culture collections under accession numbers JCM 36015^T^=DSM 118067^T^, JCM 34431^T^=DSM 118066^T^ and JCM 34432, respectively.

## 16S rRNA gene phylogeny

**Fig. 1.**
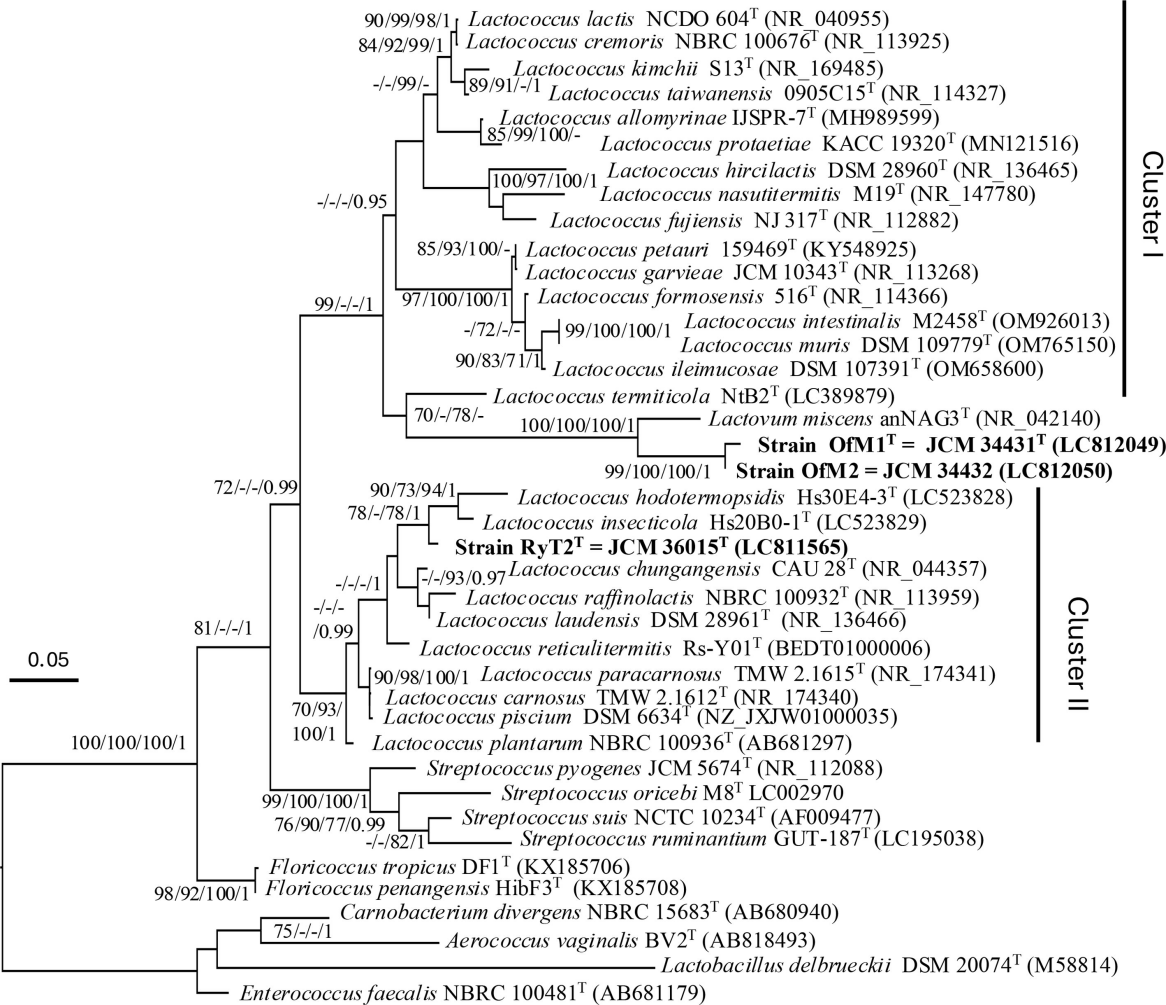
The ML phylogenetic tree with strains RyT2^T^, OfM1^T^, OfM2, type strains in the genus *Lactococcus* and the family *Streptococcaceae*, based on 16S rRNA gene sequences. The tree was inferred with unambiguously aligned nucleotide sites of 1,343 for 16S rRNA. Bootstrap values are expressed as a percentage of 1,000 replications for ML, MP and NJ analysis and 1,000,000 replications for Bayesian posterior probability and are indicated at nodes as ML/MP/NJ/PP. Values below 70% for ML and MP and 0.95 for Bayes are indicated with hyphens. The outgroup taxa in the analyses were four species in *Lactobacillales* (*Lactobacillus delbrueckii*, *Aerococcus vaginalis*, *Carnobacterium divergens* and *Enterococcus faecalis*). The accession numbers of 16S rRNA genes are given in parentheses. Bar, 0.05 substitutions per nucleotide position.

16S rRNA gene amplification was performed as described previously [4] and each PCR product was directly sequenced. The nearly full-length 16S rRNA gene sequences of the isolated strains were compared with those of 26 described species of the genus *Lactococcus* and one species of the genus *Lactovum*. All validly published species had <98% sequence similarity to these strains, and these similarities are below the limit for species (98.6%) and genus (95.0%) differentiation [18]. The strains OfM1^T^ and OfM2 showed 99.4% sequence similarity. The strain RyT2^T^ showed the highest nucleotide similarity (97.2%) to *Lactococcus chungangensis*, and OfM1^T^ and OfM2 showed the highest nucleotide similarity (94.1 and 94.6%) to *Lv. miscens*. These values were below the species- and genus-level criteria. The sequences of these strains were compared with closely related sequences in GenBank using the blastn program and aligned using the MAFFT software [19]. Phylogenetic trees were reconstructed by maximum-likelihood (ML), maximum-parsimony (MP), neighbour-joining (NJ) and Bayesian methods using RAxML-NG [20] (ML), MEGA ver. 11 [21] (MP and NJ) and MrBayes [22], respectively. The phylogenetic analysis showed that the strain RyT2^T^ forms a unique lineage among *Lactococcus* species (Figs 1 and S1, available in the online Supplementary Material). Similarly, strains OfM1^T^ and OfM2 formed a lineage together with the genus *Lactovum*. However, *Lactococcus* did not form a monophyletic clade but was divided into two clusters (designated clusters I and II) by the cluster of *Lactovum* and strains OfM1^T^ and OfM2. The strain RyT2^T^ grouped with *Lactococcus reticulitermitis* isolated from the termite *Reticulitermes speratus* in cluster II, but *L. lactis*, which was the type species of the genus *Lactococcus*, belonged to cluster I.

## Genome features

DNA extractions were performed as described previously [23]. Whole genomes of the strains were sequenced using an Illumina MiSeq instrument, generating 2×300 bp paired-end reads. After quality control, *de novo* assemblies were performed using SPAdes version 3.11.1 software [24]. The parameters of the genome assemblies were as follows: (i) genome sizes of 3,173,524, 2,309,982 and 2,267,189 bp, (ii) contig counts of 192, 49 and 18, (iii) N50 lengths of 29,322, 121,556 and 337,002, (iv) average genome coverage of 228.5, 328.1 and 131.7 for RyT2^T^, OfM1^T^ and OfM2, respectively. The resulting draft genome sequences were annotated by using the DDBJ Fast Annotation and Submission Tool (DFAST; https://dfast. ddbj.nig.ac.jphttps://dfast. ddbj.nig.ac.jp), a prokaryotic genome annotation pipeline [25]. DFAST was used to assess the completeness of the genomes, yielding results of 98.35, 94.92 and 94.92% for RyT2^T^, OfM1^T^ and OfM2, respectively. The GenBank accession numbers of strains RyT2^T^, OfM1^T^ and OfM2 are BAAGGP010000001–BAAGGP010000192, BAAGGQ010000001–BAAGGQ010000049 and BAAGGR010000001–BAAGGR010000018, respectively. Based on DFAST annotation, the genome sequences of strains RyT2^T^, OfM1^T^ and OfM2 contained 3,003, 2,169 and 2,162 Coding Sequence (CDS), 3, 5 and 6 rRNA and 46, 48 and 48 tRNA gene sequences, respectively. The G+C content of their draft genomes was 38.8, 45.6 and 46.0 mol% for strains RyT2^T^, OfM1^T^ and OfM2, respectively. The G+C content of RyT2^T^ was 38.8 mol%, similar to the closely related *Lactococcus* strains with a range of 36.9–42.2 mol%, but the G+C content of OfM1^T^ and OfM2 was significantly higher than that of *Lv. miscens* (37.60 mol%).

*In silico* prediction of the DNA–DNA hybridization (DDH) value and a pairwise average nucleotide identity (ANI) were estimated using the genome-to-genome distance calculator (GGDC) 3.0 [26] and the ANI calculator [27], respectively. The distances of each pair of genomes were inferred using formula 2, as recommended by the software vendors, which divides sequence lengths by high-scoring segment pairs. The DNA-relatedness values of strain RyT2^T^ to *L. chungangensis* CAU 28^T^ were 86.27% for ANI and 32.70% for GGDC (Table S1). These values for the most closely related type species were well below the proposed species cut-off of 95% [18] for ANI and 70% for DDH, indicating that strain RyT2^T^ represents a novel species. Strains OfM1^T^ and OfM2 shared ANI values of 95.7% and GGDC values of 63.50% with each other, whereas strains OfM1^T^ and OfM2 shared ANI values of 70.3 and 70.0% and GGDC values of 20.50 and 20.90% with *Lv. miscens* anNAG3^T^, respectively.

## Comparative genomics and phylogenomics

To clarify the systematics of the genus, we performed ANI, average amino acid identity (AAI) and core-genome phylogeny. A comprehensive analysis of genome similarity was performed by calculating ANI and AAI values of isolated strains with 26 *Lactococcus* species and *Lv. miscens* anNAG3^T^. The orthologous ANI algorithm using usearch (OrthoANIu; www.ezbiocloud.net/tools/ani) [27] and CompareM [28] software was used to calculate these values. Although the homology of 16S rRNA genes between OfM1^T^ and anNAG3^T^ (94.1%) was below the genus-level threshold (92–95%), the AAI value was within the category thresholds of the same genus (65–95%). As a result, OfM1^T^ is considered to be a new species of the genus *Lactovum*. The ANI and AAI values between *Lactococcus intestinalis* M2458^T^ and *Lactococcus muris* DSM 109779 ^T^ showed more than 95% (98.90 and 99.91%, respectively), which was above the recommended species boundary. Together with the identity of the 16S rRNA gene sequence (100%), these results indicated the close phylogenetic relationship of the two type strains. As there is clear evidence based on genomic data that both strains, M2458^T^ and DSM 109779^T^, belong to the same species, it is proposed that one name is an earlier subjective synonym of the other. *L. muris* (Afrizal *et al*. [29]) was effectively published in 2022 (validly published in the validation list no. 213 in September 2023 [30]), and *L. intestinalis* (Sun *et al*. [31]) was effectively published in 2023 (validly published in the validation list no. 216 in March 2024 [32]). Therefore, the name *L. muris* Afrizal *et al*. 2023 has priority over *L. intestinalis* Sun *et a*l. 2024. Consequently, the species *L. intestinalis* Sun *et al*. 2024 should be considered as a later heterotypic synonym of *L. muris* Afrizal *et al*. 2023.

**Fig. 2.**
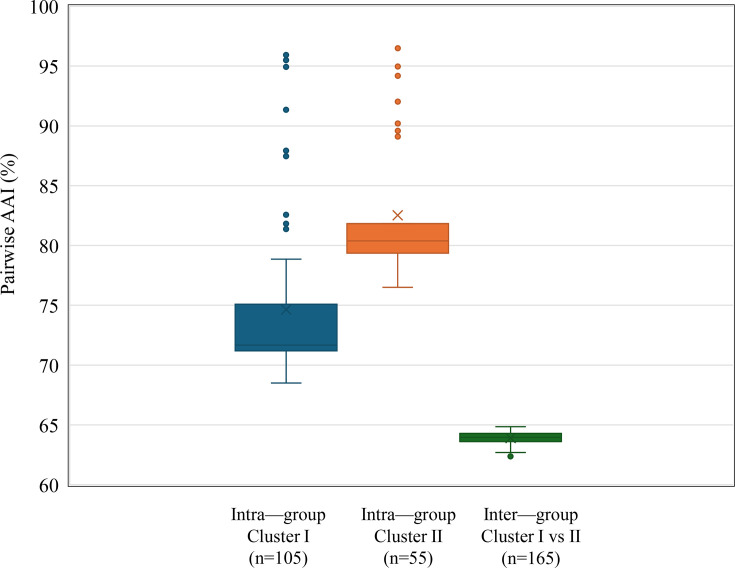
Box plot of the intra-group and inter-group AAI values in the genus *Lactococcus*. The species in each cluster are shown on the phylogenetic tree in Fig. 1.

The pairwise intra-group and inter-group distributions of AAI values are shown in Figs 2 and S2. Although the intra-group AAI values for clusters I and II were broad (68.5–95.9 and 76.5–96.5%, respectively), the inter-group AAI values between members of cluster I and cluster II were comparatively smaller than the intra-group AAI values of each cluster (62.4–64.9%). These results confirm the phylogenetic heterogeneity of *Lactococcus* and the clear separation between clusters I and II.

**Fig. 3.**
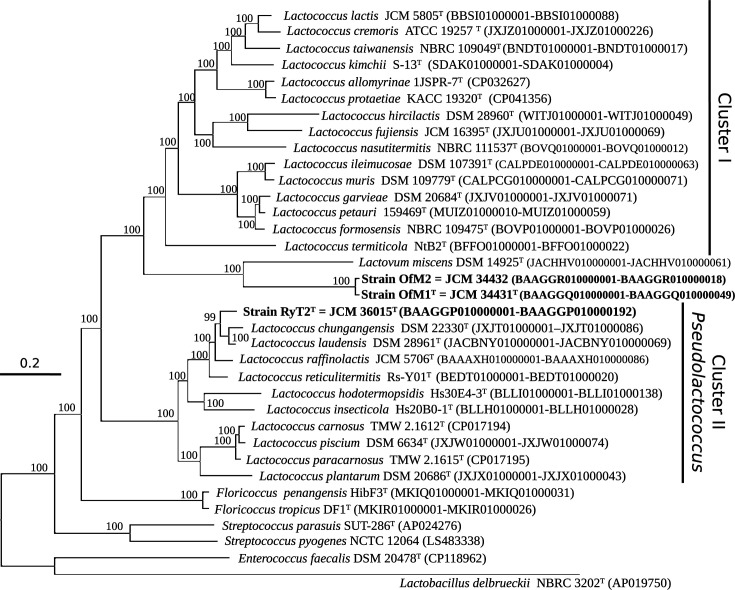
Core-genome phylogenetic tree with strains RyT2^T^, OfM1^T^, OfM2, type strains of the genus *Lactococcus* and type species of each genus in the family *Streptococcaceae*. Four species from *Lactobacillales* (*Streptococcus parasuis*, *Streptococcus pyogenes*, *Enterococcus faecalis* and *Lactobacillus delbrueckii*) were used as outgroups. The phylogenomic analysis is based on the concatenated protein sequences of 81 core genes. Bootstrap support values were calculated from 100 replicates, and only values of >80% were labelled.

From the genome sequences for type strains of all species in the genus *Lactococcus* and type species of each genus in the family *Streptococcaceae*, the up-to-date bacterial core gene set UBCG (https://www.ezbiocloud.net/tools/ubcg) [33] was used to construct a genome-based phylogenomic tree. A core-genome phylogenetic tree of all species in the genus *Lactococcus* and type species of the genera in the family *Streptococcaceae* is shown in Fig. 3. The family *Streptococcaceae* consists of four genera that do not match the cluster found in the core-genome phylogenetic analyses. However, the monophyletic separations of clusters I and II were supported by all core-genome phylogenetic analyses with 100% bootstrap values each, indicating that they formed independent taxonomic groups. Therefore, we propose a genus status for each of these two phylogenetic clusters, consisting of an emended genus *Lactococcus* (for cluster I, which includes *L. lactis*, the type species of *Lactococcus*) and *Pseudolactococcus* gen. nov., consisting of the species in cluster II. The emended genus *Lactococcus* contains 15 species, including *Lactococcus cremoris* and *Lactococcus garvieae*. The newly proposed *Pseudolactococcus* contains 11 species, including those represented by the newly isolated strain RyT2^T^. We propose *P. reticulitermitis* as the type species of the genus *Pseudolactococcus*.

## Physiology and chemotaxonomy

The morphology of the cells after 2 days of culture on trypticase soy agar (TSA; Difco) plates was observed by phase-contrast microscopy (BX-60, Olympus). To investigate physiological properties, strains RyT2^T^ and reference strains were grown on TSA, strains OfM1^T^ and OfM2 were grown on MRS and cultures were incubated anaerobically using the AnaeroPack system at 30 °C for 1–3 days. All isolated strains were able to grow under anaerobic conditions, but strain RyT2^T^ did not grow under aerobic conditions. Although strains OfM1^T^ and OfM2 showed slightly weak growth under aerobic conditions, at least on TSA and MRS media, compared to anaerobic conditions. For comparison, *L. chungangensis* JCM 17100^T^, *L. reticulitermitis* JCM 32106^T^, *Lactococcus raffinolactis* JCM 5706^T^, *Lactococcus plantarum* JCM 11056^T^, *Lactococcus piscium* JCM 16647^T^, *Lactococcus hodotermopsidis* JCM 33486^T^, *Lactococcus insecticola* JCM 33485^T^ and *Lactococcus laudensis* JCM 31965^T^ were used as reference strains. The characterization of the novel strain and the reference strains was carried out under the same conditions. To test motility, the cells were observed under the microscope. Gram staining kit (Nissui) and oxidase test strips (Nissui) were used for corresponding tests according to the manufacturer’s instructions. Catalase activity was tested using 3% (w/v) H_2_O_2_ by observing the formation of bubbles. The temperature range for growth was tested on TSA medium for strain RyT2^T^ and reference strains or MRS medium for OfM1^T^ and OfM2 incubated at 10, 20, 30, 40 and 45 °C. NaCl tolerance was tested under the various concentrations (0.5–4%, w/v). The growth pH range for the strain RyT2^T^ was determined in TSA plates with a pH range of 4–8 at 1 pH unit intervals by adjusting the HCl or NaOH. For OfM1^T^ and OfM2, the range of growth pH was determined by adjusting HCl or Na₂CO₃ at 1 pH unit intervals from pH 4 to 11 on MRS plates. After autoclaving, the pH of each plate was checked. Strain RyT2^T^ was able to grow at pH 6–8 and 1% NaCl but not at pH 5 and 2% NaCl. On the other hand, strains OfM1^T^ and OfM2 were able to grow at pH 5–8 and 1% NaCl but not above pH 9 and 2% NaCl. The optimum growth pH was pH 7–8 for three strains. Strain RyT2^T^ grew at temperatures from 10 to 30 °C, with optimum growth at 30 °C. Strains OfM1^T^ and OfM2 grew at temperatures from 20 to 35 °C with optimum growth at 30 °C. The optimal growth temperatures and pH of strains OfM1^T^ and OfM2 were similar to *Lv. miscens*. Whereas *Lv. miscens* had the growth ability of cold resistance (0–35 °C) and low pH tolerance (pH 3.5–7.5) [12]. These isolated strains were non-motile, Gram-stain-positive and catalase-negative. The results of the temperature, pH and NaCl tests are shown in Table 1 in comparison with those of other type strains of the genus *Lactococcus*.

**Table 1.** Differential characteristics of strain RyT2^T^, OfM1^T^, OfM2 and related species of the genus *Lactococcus* Strains: 1, RyT2^T^; 2, *L. chungangensis* JCM 17100^T^; 3, *L. laudensis* JCM 31965^T^; 4, *L. raffinolactis* JCM 5706^T^; 5, *L. insecticola* JCM 33485^T^; 6, *L. hodotermopsidis* JCM 33486^T^; 7, *L. reticulitermitis* JCM 32106^T^; 8, *L. piscium* JCM 16647^T^; 9, *L. plantarum* JCM 11056^T^; 10, OfM1^T^; 11, OfM2; 12, *Lv. miscens* DSM 14925^T^. Data were obtained in this study unless otherwise mentioned. Data for *Lv. miscens* DSM 14925^T^ were obtained from Matthies *et al*. [12]. +, Positive; −, negative; w, weakly positive; na, not available.

Characteristic	1	2	3	4	5	6	7	8	9	10	11	12
Cell morphology	Rod	Coccoid or ovoid	Coccoid	Coccoid	Cocco-bacilli	Cocco-bacilli	Cocco-bacilli	Short rods to ovoid	Coccoid or ovoid	Short-rod	Short-rod	Ovoid
Peptidoglycan type	A3a Lys-Thr-Ala	A3a Lys-Ala_2_	A3a Lys-Thr-Ala^*a*^	A3a Lys-Thr-Ala^*a*^	A4a Lys-Asp^*b*^	A3a Lys-Thr-Ala^*b*^	A3a Lys-Ala_1-2_^*a*^	A3a Lys-Ala_1-2_^*a*^	A3a Lys-Thr-Ala^*a*^	A3a Lys-Thr-Ala	A3a Lys-Thr-Ala	na
G+C (mol%)	38.8	40.0^*c*^	41.1^*c*^	41.5^*d*^	42.2^*b*^	39.3^*b*^	41.3^*a*^	38.5^*e*^	36.9–38.1^*f*^	45.6	46.0	37.6
Growth at:												
10 °C	w	+	+	+	+	−	w	w	+	−	−	+
30 °C	+	+	+	+	+	+	+	+	+	+	+	+
40 °C	−	−	−	−	−	−	−	−	−	−	−	−
pH 5.0	−	+	+	+	+	−	+	−	+	+	+	+
Growth in 1% (w/v) NaCl	+	+	+	+	+	+	+	+	+	+	+	na
Growth in 2% (w/v) NaCl	−	+	+	+	+	+	+	+	+	−	−	na
Acid production from:												
Erythritol	−	−	−	±	−	−	−	−	−	−	−	na
d-Ribose	−	−	＋	−	−	−	−	−	−	−	−	na
d-Xylose	**＋**	−	＋	＋	−	±	−	−	−	−	−	na
d-Galactose	−	−	±	＋	−	−	＋	±	−	−	−	＋
d-Glucose	＋	＋	＋	＋	−	＋	＋	＋	＋	＋	＋	＋
d-Fructose	＋	＋	＋	＋	−	＋	＋	＋	＋	±	±	＋
d-Mannose	＋	＋	＋	＋	−	＋	＋	＋	＋	＋	＋	na
d-Mannitol	＋	+	＋	＋	−	−	−	−	＋	−	−	＋
d-Sorbitol	±	−	＋	−	−	−	＋	−	＋	−	−	na
Methyl *α*-d-glucopyranoside	−	−	±	±	−	−	−	−	＋	−	−	na
*N*-Acetyl glucosamine	＋	＋	＋	＋	−	±	＋	±	＋	−	−	na
Amygdalin	＋	+	＋	±	−	±	＋	−	＋	−	−	na
Arbutin	＋	±	＋	＋	−	−	＋	−	＋	−	−	na
Salicin	＋	+	＋	＋	−	±	＋	±	＋	−	−	na
d-Cellobiose	＋	+	＋	＋	−	±	＋	−	＋	−	−	＋
d-Maltose	−	+	＋	＋	−	−	＋	−	＋	−	−	＋
d-Lactose	−	−	＋	＋	−	−	±	＋	−	−	−	na
d-Melibiose	−	−	−	＋	−	−	−	±	±	−	−	na
d-Sucrose	±	+	＋	＋	−	−	−	±	＋	−	−	na
d-Trehalose	＋	+	＋	＋	−	±	＋	−	＋	−	−	na
Inulin	−	−	＋	−	−	−	−	−	＋	−	−	na
d-Melezitose	−	−	＋	−	−	−	−	−	＋	−	−	na
d-Rafinose	−	−	−	＋	−	−	−	±	−	−	−	na
Starch	−	−	＋	−	−	−	＋	−	−	−	−	na
Glycogen	−	−	＋	−	−	−	＋	−	−	−	−	na
Gentiobiose	＋	+	＋	±	−	±	＋	−	＋	−	−	na
d-Turanose	−	+	＋	−	−	−	−	−	＋	−	−	na
d-Tagatose	−	−	−	−	−	−	＋	−	−	−	−	na
Gluconate	±	−	−	−	−	−	−	−	−	−	−	na
Enzyme activities												
Alkaline phosphatase	−	−	−	−	−	＋	−	−	−	＋	−	na
Esterase	−	＋	＋	＋	−	−	＋	−	＋	−	−	na
Leucine-arylamidase	＋	＋	＋	＋	＋	＋	＋	＋	＋	＋	＋	na
Chymotrypsin	−	−	w	−	−	−	w	w	−	−	−	na
Acid phosphatase	＋	＋	＋	＋	−	−	＋	＋	＋	＋	＋	na
Naphthol-AS-BI-phosphohydrolase	−	w	＋	＋	w	−	＋	w	＋	＋	＋	na
*α*-Galactosidase	−	−	−	w	−	w	−	−	−	−	−	na
*ß*-Galactosidase	−	−	−	−	−	＋	−	−	−	＋	−	na
*α*-Glucosidase	−	＋	＋	＋	−	−	＋	−	＋	＋	＋	na
*ß*-Glucosidase	＋	＋	＋	＋	＋	＋	＋	＋	＋	−	−	na
Degradation activity												
Starch	+	+	+	+	+	+	+	+	+	+	+	na
Xylan	−	−	+	+	−	+	+	+	+	+	−	na
CMC	−	−	−	−	−	−	−	−	−	−	−	na
Pectin	−	−	−	−	−	−	−	−	−	−	−	na

Part of the data for DNA G+C content and peptidoglycan type were collected from the literature: *a*, Yuki *et al*. [5]; *b*, Noda *et al*. [10]; *c*, Meucci *et al*. [3]; *d*, Garvie [40]; *e*, Williams *et al*. [36]; *f*, Collins *et al*. [39].

Acid production from carbohydrates was tested using the API 50 CHL fermentation kit according to the manufacturer’s instructions. API ZYM (bioMérieux) was used according to the manufacturer’s instructions. The results of three isolated strains differed from those of the reference strains (Table 1). *L. insecticola* could not produce acids from various substrates contained in API 50CH test strips as previously reported [10]. The ability to degrade and ferment different polysaccharides, including 1% xylan (from beech wood; Apollo Scientific), 1% starch (Wako), 1% pectin (from citrus; Nacalai) or 1% carboxymethyl cellulose (CMC; Nacalai), was tested using peptone-yeast extract broth for 3 days, and the decrease in pH due to acid production was then recorded according to the method described previously [4]. Strain RyT2^T^ degraded starch, strain OfM1^T^ degraded starch and xylan, but strain OfM2 only degraded starch. l- or d-lactic acid isomers were identified using d-/l-lactic acid test kits (Roche Diagnostics) according to the manufacturer’s instructions. The type of lactic acid fermentation was examined by culturing the strains in TS broth at 30 °C for 2 days under anaerobic conditions. Three isolated strains produced l-lactic acid (99%).

The fatty acid methyl esters of three isolated strains and the reference strains were determined according to the method described previously [34]. Fatty acid methyl esters were obtained from cells grown on TSA plates for RyT2^T^ or MRS plates for OfM1^T^ and OfM2 at 30 °C for 3 days under anaerobic conditions by saponification, methylation and extraction using minor modifications [35] of the methods of Miller [34]. Cellular fatty acid profiles were determined according to version 6.2B of the Sherlock Microbial Identification System (MIDI) and using the TSBA 6 database. The predominant cellular fatty acids of strain RyT2^T^ were C_16:0_ (32.5%), summed feature 4 (including C_17:1_ iso I/anteiso B; 16.0%) and summed feature 8 (comprising C_18:1_* ω*7*c*/C_18:1_* ω*6*c*; 26.2%). The predominant cellular fatty acids of strain OfM1^T^ were C_14:0_ (19.7%), C_16:0_ (30.9%) and summed feature 4 (15.2%). The predominant cellular fatty acids of strain OfM2 were C_16:0_ (22.9%), C_18:1_* ω*9*c* (13.3%) and feature 8 (comprising C_18:1_* ω*7*c*/C_18:1_* ω*6*c*; 22.8%). Although 10-methyl branched fatty acid is not a common fatty acid in *Lactococcus*, it has been detected in *L. insecticola*, *L. plantarum* and *L. piscium*, as previously reported [10,36]. The fatty acid profile of both strains OfM1^T^ and OfM2 differed from that of the *Lactococcus* reference strains, and the complete cellular fatty acid compositions of the three isolated strains and the reference species are given in Table 2. The amino acid composition of the cell wall peptidoglycan was determined as described by Komagata and Suzuki [37]. The data indicated that the cell wall peptidoglycan type of strains RyT2^T^, OfM1^T^ and OfM2 was A3a, Lys-Thr-Ala. The peptidoglycan type of *L. chungangensis* was determined in this study as A3a, Lys-Ala_2_. The peptidoglycan types of other reference strains have been reported as A3a, Lys-Ala_1-2_ (*L. reticulitermitis* and *L. piscium*) [5], A3a, Lys-Thr-Ala (*L. laudensis*, *L. raffinoactis*, *L. hodotermopsidis* and *L. plantarum*) [10] or A4a, Lys-Asp (*L. insecticola*) [10]. Based on phenotypic characteristics, phylogenetic positions and genomic compositions, strains RyT2^T^ and OfM1^T^ should represent novel species of the genera *Pseudolactococcus* or *Lactovum*, for which we proposed the names *Pseudolactococcus yaeyamensis* sp. nov. and *Lactovum odontotermitis* sp. nov., respectively.

**Table 2.** Cellular fatty acid compositions of strains RyT2^T^, OfM1^T^ and OfM2 and related species of the genus *Lactococcus* Strains: 1, RyT2^T^; 2, *L. chungangensis* JCM 17100^T^; 3, *L. laudensis* JCM 31965^T^; 4, *L. raffinolactis* JCM 5706^T^; 5, *L. insecticola* JCM 33485^T^; 6, *L. hodotermopsidis* JCM 33486^T^; 7, *L. reticulitermitis* JCM 32106^T^; 8, *L. piscium* JCM 16647^T^; 9, *L. plantarum* JCM 11056^T^; 10, OfM1^T^; 11, OfM2. –, Not detected or <1.0% of the total fatty acids.

Fatty acid	1	2	3	4	5	6	7	8	9	10	11
C_12 : 0_	1.4	2.3	1.0	1.4	2.1	**17.6**	2.1	2.5	2.2	–	–
C_14 : 0_	9.9	**23.3**	8.5	**17.9**	**22.0**	**14.2**	**19.8**	**25.7**	**15.7**	**19.7**	8.1
C_16 : 0_	**32.5**	**27.7**	**40.8**	**25.5**	**25.2**	**20.2**	**26.4**	**22.8**	**32.8**	**30.9**	**22.9**
C_18 : 0_	2.3	1.1	4.3	–	–	2.9	–	–	–	–	1.0
10-methyl-C_19 : 0_	–	–	–	–	8.1	–	–	7.6	4.6	9.9	5.5
cyclo-C_17:0_	–	–	–	–	–	–	–	2.7	1.5	–	–
cyclo-C_19:0_* ω*8*c*	–	–	–	–	**12.5**	–	–	**26.4**	**22.5**	6.8	2.1
C_16:1_* ω*9*c*	2.7	1.5	2.4	1.6	–	–	2.7	3.7	1.4	–	–
C_16:1_* ω*5*c*	1.0	1.5	2.0	1.9	–	–	2.0	1.3	–	–	–
C_17:1_* ω*7*c*	–	–	–	–	–	–	–	–	–	–	1.8
C_17:1_* ω*8*c*	4.1	5.0	2.3	5.3	1.1	3.0	2.9	–	–	1.0	–
C_17:1_* ω*9*c*	–	–	–	–	–	–	–	–	–	–	8.6
C_18:1_* ω*9*c*	–	–	–	–	–	–	–	–	–	9.0	**13.3**
iso-C_17:1_* ω*10*c*	–	–	–	1.0	–	–	–	–−	–	–	–
C_19:1_ iso I	1.0	–	–	–	–	–	–	–	–	–	2.6
anteiso-C_17:1_* ω*9*c*	1.8	–	–	–	–	–	–	–	–	–	–
Summed feature 1	–	–	–	–	3.1	4.1	–	–	–	–	–
Summed feature 3	1.2	1.8	1.6	2.5	1.5	–	5.3	3.9	2.2	2.8	1.6
Summed feature 4	**14.5**	**15.5**	3.3	**15.0**	**18.7**	**22.5**	5.3	4.9	6.7	3.2	3.2
Summed feature 7	–	–	–	–	–	–	–	–	–	4.4	4.5
Summed feature 8	**26.2**	**18.8**	**31.6**	**25.0**	2.9	**12.4**	**29.8**	4.2	6.5	8.8	**22.8**

Major components (>10%) are highlighted in bold.

*Summed features are fatty acids that cannot be resolved reliably from another fatty acid using the chromatographic conditions chosen. The MIDI system groups these fatty acids together as one feature with a single percentage of the total. Summed feature 1 contains C_15:1_ iso H/C_13:0_ 3OH; summed feature 3 contains C_16:1_* ω*7*c*/C_16:1_* ω*6*c*; summed feature 4 contains C_17:1_ iso I/anteiso B; summed feature 7 contains C_19:1_*ω*7*c* and/or C_19:1_*ω*6*c* and/or C_19:1_ 19cy; summed feature 8 comprises C_18:1_* ω*7*c*/C_18:1_* ω*6*c*.

## Description of *Pseudolactococcus* gen. nov.

*Pseudolactococcus* (Pseu.do.lac.to.coc’cus. Gr. neut. adj. *pseudes*, false; N.L. masc. n. *Lactococcus*, a bacterial genus name; N.L. masc. n. *Pseudolactococcus*, false *Lactococcus*).

Gram-stain-positive, catalase-negative, heterofermentative. Cell morphology is variable, coccoid to rod. Most strains grow at 10 °C, and the optimal growth pH is typically observed at pH 6.0–8.0. The G+C content of DNA ranges from 36.9 to 42.2 mol%. Strains in the genus were isolated from silage, fermented vegetables, fish and termites. *Pseudolactococcus* species generally metabolize a broad spectrum of hexoses and disaccharides. A phylogenetic tree on the basis of 16S rRNA genes of all species in the genus *Pseudolactococcus* is shown in Fig. 1.

The type species of the genus is *P. reticulitermitis*.

## Description of *Pseudolactococcus yaeyamensis* sp. nov.

*Pseudolactococcus yaeyamensis* (yae.yam.en’sis. N.L. masc. adj. *yaeyamensis*, pertaining to the Yaeyama Islands).

Cells are non-motile, Gram-stain-positive, catalase-negative, anaerobic and rod-shaped, 1.81±0.27–0.47±0.08 mm in size. It grows at 10–30 ˚C (optimum, 30 ˚C) with 0.5–1% (w/v) NaCl, at pH 6.0–8.0. The main metabolic end product is l-lactic acid (99:1). In the API 50 CHL fermentation kit, acid is produced from d-xylose, d-glucose, d-fructose, d-mannose, d-mannitol, *N*-acetyl glucosamine, amygdalin, arbutin, aesculin ferric citrate, salicin, d-cellobiose, d-trehalose and gentiobiose, and acid is weakly produced from d-sorbitol, d-sucrose and gluconate. Possesses active leucine-arylamidase, acid phosphatase and *ß*-glucosidase. The major cellular fatty acids (>10%) are C_16:0_, summed feature 4 (C_17:1_ iso I/anteiso B) and summed feature 8 (C_18:1_* ω*7*c*/C_18:1_* ω*6*c*). The cell wall peptidoglycan is type A3a, Lys-Thr-Ala. The type strain, RyT2^T^ (=JCM 36015^T^=DSM 118067^T^), was isolated from the gut of the wood-feeding lower termite *R. yaeyamanus*. The genomic DNA G+C content of the type strain, determined on the basis of the draft genome sequence, is 38.8 mol%. The GenBank/EMBL/DDBJ accession number for the 16S rRNA gene sequence of the type strain is LC811565 and the numbers for the whole genome sequence are BAAGGP010000001–BAAGGP010000192.

## Description of *Pseudolactococcus carnosus* comb. nov.

*Pseudolactococcus carnosus* (car.no’sus. L. masc. adj. *carnosus*, pertaining to flesh).

Basonym: *Lactococcus carnosus* Hilgarth *et al.* 2020.

Characteristics of the species are as described [38]. The G+C content of DNA is 38.8 mol%. Isolated from high-oxygen modified-atmosphere packaged beef steaks.

The type strain is TMW 2.1612^T^ (=CECT 30115^T^=DSM 111016^T^).

Genome sequence accession number: CP017194.

16S rRNA gene accession number: MT772277, NR_174340.

## Description of *Pseudolactococcus chungangensis* comb. nov.

*Pseudolactococcus chungangensis* (chung.ang.en’sis. N.L. masc. adj. *chungangensis*, named after Chungang University, where taxonomic studies on this species were performed).

Basonym: *Lactococcus chungangensis* Cho *et al.* 2008.

Characteristics of the species are as described [1]. The G+C content of DNA is 40.0 mol%. Isolated from activated sludge wastewater treatment plant.

The type strain is CAU 28^T^ (=CCUG 55099^T^=DSM 22330^T^=JCM 17100^T^=KCTC 13185^T^).

Genome sequence accession number: JXJT01000001–JXJT01000086.

16S rRNA gene accession number: EF694028, NR_044357.

## Description of *Pseudolactococcus hodotermopsidis* comb. nov.

*Pseudolactococcus hodotermopsidis* (ho.do.ter.mop’si.dis. N.L. masc. n. *Hodotermopsis*, a termite genus. N.L. gen. n. *hodotermopsidis*, of *Hodotermopsis*, referring to the genus name of the termite).

Basonym: *Lactococcus hodotermopsidis* Noda *et al.* 2020.

Characteristics of the species are as described [10]. The G+C content of DNA is 39.3 mol%. Isolated from the gut of the wood-feeding lower termite *Hodotermopsis sjostedti*.

The type strain is Hs30E4-3^T^ (=DSM 110148^T^=JCM 33486^T^).

Genome sequence accession number: BLLI01000001–BLLI01000138.

16S rRNA gene accession number: LC523828.

## Description of *Pseudolactococcus insecticola* comb. nov.

*Pseudolactococcu*s *insecticola* (in.sec.ti’co.la. L. past part. *insectus*, divided into sections; N.L. neut. n. *insectum*, insect; L. suff. -*cola*, from L. masc. or fem. n. *incola*, inhabitant; N.L. masc. n. *insecticola*, inhabitant of insects).

Basonym: *Lactococcus insecticola* Noda *et al.* 2020.

Characteristics of the species are as described [10]. The G+C content of DNA is 42.2 mol%. Isolated from the gut of the wood-feeding lower termite *H. sjostedti*.

The type strain is Hs20B0-1^T^ (=DSM 110147^T^=JCM 33485^T^).

Genome sequence accession number: BLLH01000001–BLLH01000028.

16S rRNA gene accession number: LC523829.

## Description of *Pseudolactococcus laudensis* comb. nov.

*Pseudolactococcus laudensis* (laud.en’sis. L. masc. adj. *laudensis*, pertaining to Lodi (Italy), named *Laus Pompeia* in Roman times, where the species was isolated).

Basonym: *Lactococcus laudensis* Meucci *et al.* 2015.

Characteristics of the species are as described [3]. The G+C content of DNA is 41.1 mol%. Isolated from cow milk.

The type strain is 4195^T^ (=DSM 28961^T^=JCM 31965^T^=LMG 28353^T^).

Genome sequence accession number: JACBNY010000001–JACBNY010000069.

16S rRNA gene accession number: KJ394457, NR_136466.

## Description of *Pseudolactococcus paracarnosus* comb. nov.

*Pseudolactococcus paracarnosus* (pa.ra.car.no’sus. Gr. prep. *para*, beside; L. masc. adj. *carnosus*, pertaining to flesh; N.L. masc. adj. *paracarnosus*, beside *carnosus*, referring to the close phylogenetic and habitat relationship to *Lactococcus carnosus*).

Basonym: *Lactococcus paracarnosus* Hilgarth *et al.* 2020.

Characteristics of the species are as described [38]. The G+C content of DNA is 38.2 mol%. Isolated from high-oxygen modified-atmosphere packaged beef steaks.

The type strain is TMW 2.1615^T^ (=CECT 30116^T^=DSM 111017^T^).

Genome sequence accession number: CP017195.

16S rRNA gene accession number: MT772278, NR_174341.

## Description of *Pseudolactococcus piscium* comb. nov.

*Pseudolactococcus piscium* (pis’ci.um. L. gen. pl. n. *piscium*, of fishes).

Basonym: *Lactococcus piscium* Williams *et al.* 1990.

Characteristics of the species are as described [36]. The G+C content of DNA is 38.5 mol%. Isolated from salmonid fish.

The type strain is HR1A-68^T^ (=DSM 6634^T^=JCM 16647^T^=NCFB 2778^T^).

Genome sequence accession number: JXJW01000001–JXJW01000074.

16S rRNA gene accession number: NZ_JXJW01000035.

## Description of *Pseudolactococcus plantarum* comb. nov.

*Pseudolactococcus plantarum* (plan.ta’rum. L. fem. n. *planta*, any vegetable production that serves to propagate the species, a plant; L. gen. fem. pl. n. *plantarum*, of plants).

Basonym: *Streptococcus plantarum* Collins *et al*. 1984.

Characteristics of the species are as described [39]. The G+C content of DNA is 36.9–38.1 mol%. Isolated from frozen peas.

The type strain is NCDO 1869^T^ (=DSM 20686^T^=JCM 11056^T^=NBRC 100936^T^).

Genome sequence accession number: JXJX01000001–JXJX01000043.

16S rRNA gene accession number: AB681297.

## Description of *Pseudolactococcus raffinolactis* comb. nov.

*Pseudolactococcus raffinolactis* (raf.fi.no.lac’tis. N.L. neut. n. *raffinosum*, raffinose; L. neut. n. lac, milk; N.L. gen. n. *raffinolactis*, raffinose fermenting bacterium from milk).

Basonym: *Streptococcus raffinolactis* Orla-Jensen and Hansen 1932 (Approved Lists 1980).

Characteristics of the species are as described [40]. The G+C content of DNA is 41.5 mol%. Isolated from milk.

The type strain is NCDO 617^T^ (=DSM 20443^T^=JCM 5706^T^ =NBRC 100932^T^).

Genome sequence number: BAAAXH010000001–BAAAXH010000086.

16S rRNA gene accession number: NR_113959.

## Description of *Pseudolactococcus reticulitermitis* comb. nov.

*Pseudolactococcus reticulitermitis* (re.ti.cu.li.ter’mi.tis. N.L. gen. n. *reticulitermitis*, of *Reticulitermes*, the genus of the termite from which the type strain was isolated).

Basonym: *Lactococcus reticulitermitis* Yuki *et al.* 2018.

Characteristics of the species are as described [5]. The G+C content of DNA is 41.3 mol%. Isolated from the gut of the wood-feeding lower termite *R. speratus*.

The type strain is Rs-Y01^T^ (=DSM 105715^T^=JCM 32106^T^).

Genome sequence accession number: BEDT01000001–BEDT01000020.

16S rRNA gene accession number: BEDT01000006.

## Description of *Lactovum odontotermitis* sp. nov.

*Lactovum odontotermitis* (o.don.to.ter’mi.tis. N.L. masc. n. *Odontotermes*, the scientific name of a genus of termite; N.L. gen. n. *odontotermitis*, of a termite of the genus *Odontotermes*, referring to the isolation of the type strain from the termite *O. formosanus*).

Cells are non-motile, Gram-stain-positive, catalase-negative, anaerobic and short rod-shaped, 1.52±0.20–0.84±0.15 mm in size. Colonies on MRS agar plates after 3 days of incubation at 30 °C under anaerobic conditions are 1–2 mm in diameter, cream coloured, circular, entire and smooth. It grows at 20–35 °C with 1.0% (w/v) NaCl at pH 5.0–8.0 but not at 40 °C under pH 4.0 and over pH 9.0. The main metabolic end product is l-lactic acid (99:1). Ferments soluble starch and xylan. In the API 50 CHL fermentation kit, acid is produced from d-glucose, d-mannose and aesculin ferric citrate; it is weakly produced from d-fructose. Possesses active alkaline phosphatase, leucine-arylamidase, acid phosphatase, naphthol-AS-BI-phosphohydrolase, *ß*-galactosidase and *α*-glucosidase. The major cellular fatty acids are C_14:0_ and C_16:0_. The cell wall peptidoglycan is type A3a, Lys-Thr-Ala. The type strain, OfM1^T^ (=JCM 34431^T^=DSM 118066^T^), was isolated from the gut of the wood-feeding lower termite *O. formosanus*. The genomic DNA G+C content of the type strain, determined on the basis of the draft genome sequence, is 45.6 mol%. One additional strain OfM2 (=JCM 34432) is included in this species. The GenBank/EMBL/DDBJ accession number for the 16S rRNA gene sequence of the type strain is LC812049 and the numbers for the whole genome sequence are BAAGGQ010000001–BAAGGQ010000049.

## Supplementary material

10.1099/ijsem.0.006803 Uncited Supplementary Material 1.

## Abbreviations:

AAI: average amino acid identity
ANI: average nucleotide identity
CDS: Coding sequence
CMC: carboxymethyl cellulose
DDH: DNA–DNA hybridization
DFAST: DDBJ Fast Annotation and Submission Tool
GGDC: genome-to-genome distance calculator
ML: maximum-likelihood
MP: maximum-parsimony
MRS: de Man–Rogosa–Sharpe
NJ: neighbour-joining
TS: trypticase soy
TSA: trypticase soy agar

## References

[1] Cho S-L, Nam S-W, Yoon J-H, Lee J-S, Sukhoom A (2008). *Lactococcus chungangensis* sp. nov., a lactic acid bacterium isolated from activated sludge foam. Int J Syst Evol Microbiol.

[2] Chen Y-S, Otoguro M, Lin Y-H, Pan S-F, Ji S-H (2014). *Lactococcus formosensis* sp. nov., a lactic acid bacterium isolated from yan-tsai-shin (fermented broccoli stems). Int J Syst Evol Microbiol.

[3] Meucci A, Zago M, Rossetti L, Fornasari ME, Bonvini B (2015). *Lactococcus hircilactis* sp. nov. and *Lactococcus laudensis* sp. nov., isolated from milk. Int J Syst Evol Microbiol.

[4] Noda S, Sakamoto M, Aihara C, Yuki M, Katsuhara M (2018). *Lactococcus termiticola* sp. nov., isolated from the gut of the wood-feeding higher termite *Nasutitermes takasagoensis*. Int J Syst Evol Microbiol.

[5] Yuki M, Sakamoto M, Nishimura Y, Ohkuma M (2018). *Lactococcus reticulitermitis* sp. nov., isolated from the gut of the subterranean termite *Reticulitermes speratus*. Int J Syst Evol Microbiol.

[6] Goodman LB, Lawton MR, Franklin-Guild RJ, Anderson RR, Schaan L (2017). *Lactococcus petauri* sp. nov., isolated from an abscess of a sugar glider. Int J Syst Evol Microbiol.

[7] Pérez T, Balcázar JL, Peix A, Valverde A, Velázquez E (2011). *Lactococcus lactis* subsp. tructae subsp. nov. isolated from the intestinal mucus of brown trout (*Salmo trutta*) and rainbow trout (*Oncorhynchus mykiss*). Int J Syst Evol Microbiol.

[8] Heo J, Cho H, Tamura T, Saitou S, Park K (2019). *Lactococcus allomyrinae* sp. nov., isolated from gut of larvae of *Allomyrina dichotoma*. Int J Syst Evol Microbiol.

[9] Wang XM, Ma S, Yang SY, Peng R, Zheng Y (2016). *Paenibacillus nasutitermitis* sp. nov., isolated from a termite gut. Int J Syst Evol Microbiol.

[10] Noda S, Koyama F, Aihara C, Ikeyama N, Yuki M (2020). *Lactococcus insecticola* sp. nov. and *Lactococcus hodotermopsidis* sp. nov., isolated from the gut of the wood-feeding lower termite *Hodotermopsis sjostedti*. Int J Syst Evol Microbiol.

[11] Bauer S, Tholen A, Overmann J, Brune A (2000). Characterization of abundance and diversity of lactic acid bacteria in the hindgut of wood- and soil-feeding termites by molecular and culture-dependent techniques. Arch Microbiol.

[12] Matthies C, Gössner A, Acker G, Schramm A, Drake HL (2004). *Lactovum miscens* gen. nov., sp. nov., an aerotolerant, psychrotolerant, mixed-fermentative anaerobe from acidic forest soil. Res Microbiol.

[13] Kikuchi Y, Hayatsu M, Hosokawa T, Nagayama A, Tago K (2012). Symbiont-mediated insecticide resistance. Proc Natl Acad Sci USA.

[14] Brune A, Ohkuma M, Bignell DE, Roisin Y, Lo N N (2011). Biology of Termites: A Modern Synthesis.

[15] Brune A (2014). Symbiotic digestion of lignocellulose in termite guts. Nat Rev Microbiol.

[16] Noda S, Shimizu D, Yuki M, Kitade O, Ohkuma M (2018). Host-symbiont cospeciation of termite-gut cellulolytic protists of the genera *Teranympha* and *Eucomonympha* and their *Treponema* endosymbionts. Microbes Environ.

[17] Ohbayashi T, Itoh H, Lachat J, Kikuchi Y, Mergaert P (2019). *Burkholderia* gut symbionts associated with European and Japanese populations of the dock bug *Coreus marginatus* (Coreoidea: Coreidae). Microbes Environ.

[18] Konstantinidis KT, Rosselló-Móra R, Amann R (2017). Uncultivated microbes in need of their own taxonomy. ISME J.

[19] Katoh K, Standley DM (2013). MAFFT multiple sequence alignment software version 7: improvements in performance and usability. Mol Biol Evol.

[20] Stamatakis A (2014). RAxML version 8: a tool for phylogenetic analysis and post-analysis of large phylogenies. Bioinformatics.

[21] Stecher G, Tamura K, Kumar S (2020). Molecular Evolutionary Genetics Analysis (MEGA) for macOS. Mol Biol Evol.

[22] Ronquist F, Teslenko M, van der Mark P, Ayres DL, Darling A (2012). MrBayes 3.2: efficient Bayesian phylogenetic inference and model choice across a large model space. Syst Biol.

[23] Noda S, Aihara C, Yuki M, Ohkuma M (2018). Draft genome sequence of *Lactococcus* sp. strain NtB2 (JCM 32569), isolated from the gut of the higher termite *Nasutitermes takasagoensis*. Genome Announc.

[24] Bankevich A, Nurk S, Antipov D, Gurevich AA, Dvorkin M (2012). SPAdes: a new genome assembly algorithm and its applications to single-cell sequencing. J Comput Biol.

[25] Tanizawa Y, Fujisawa T, Kaminuma E, Nakamura Y, Arita M (2016). DFAST and DAGA: web-based integrated genome annotation tools and resources. Biosci Microbiota Food Health.

[26] Meier-Kolthoff JP, Carbasse JS, Peinado-Olarte RL, Göker M (2022). TYGS and LPSN: a database tandem for fast and reliable genome-based classification and nomenclature of prokaryotes. *Nucleic Acids Res*.

[27] Yoon SH, Ha SM, Lim JM, Kwon SJ, Chun J (2017). A large-scale evaluation of algorithms to calculate average nucleotide identity. Antonie van Leeuwenhoek.

[28] Parks D (2020). CompareM: a toolbox for comparative genomics. https://github.com/dparks1134/CompareM.

[29] Afrizal A, Jennings SAV, Hitch TCA, Riedel T, Basic M (2022). Enhanced cultured diversity of the mouse gut microbiota enables custom-made synthetic communities. Cell Host Microbe.

[30] Oren A, Göker M (2023). Validation List no. 213. Valid publication of new names and new combinations effectively published outside the IJSEM. Int J Syst Evol Microbiol.

[31] Sun P, Li X, Shi W, Zhang L, Li M (2023). *Lactococcus intestinalis* sp. nov., a new lactic acid bacterium isolated from intestinal contents in Alzheimer’s disease mice. Antonie van Leeuwenhoek.

[32] Oren A, Göker M (2024). Validation List no. 216. Valid publication of new names and new combinations effectively published outside the IJSEM. Int J Syst Evol Microbiol.

[33] Kim J, Na S-I, Kim D, Chun J (2021). UBCG2: Up-to-date bacterial core genes and pipeline for phylogenomic analysis. J Microbiol.

[34] Miller LT (1982). Single derivatization method for routine analysis of bacterial whole-cell fatty acid methyl esters, including hydroxy acids. J Clin Microbiol.

[35] Kuykendall LD, Roy MA, O’neill JJ, Devine TE (1988). Fatty acids, antibiotic resistance, and deoxyribonucleic acid homology groups of *Bradyrhizobium japonicum*. Int J Syst Bacteriol.

[36] Williams AM, Fryer JL, Collins MD (1990). *Lactococcus piscium* sp. nov. a new *Lactococcus* species from salmonid fish. FEMS Microbiol Lett.

[37] Komagata K, Suzuki K (1987). Lipid and cell-wall analysis in bacterial systematics. Methods Microbiol.

[38] Hilgarth M, Werum V, Vogel RF (2020). *Lactococcus carnosus* sp. nov. and *Lactococcus paracarnosus* sp. nov., two novel species isolated from modified-atmosphere packaged beef steaks. Int J Syst Evol Microbiol.

[39] Collins MD, Farrow JA, Phillips BA, Kandler O (1983). *Streptococcus garvieae* sp. nov. and *Streptococcus plantarum* sp. nov. J Gen Microbiol.

[40] Garvie EI (1978). *Streptococcus raffinolactis* Orla-Jensen Hansen, a group *Streptococcus* found in raw milk. Int J Syst Bacteriol.

