# Supplementary material for: Genome-based reclassification of the genus Lactococcus and two novel species Pseudolactococcus yaeyamensis gen. nov., sp. nov. and Lactovum odontotermitis sp. nov. isolated from the gut of termites
Source: Int J Syst Evol Microbiol. 2025 Jun 4;75(6):006803. doi: 10.1099/ijsem.0.006803 (PMC12281737; doi:10.1099/ijsem.0.006803)
Supplement: Uncited Supplementary Material 1. [file ijsem-75-06803-s001.pdf]

Genome-based reclassification of the genus *Lactococcus* and two novel species *Pseudolactococcus yaeyamensis* gen. nov., sp. nov. and *Lactovum odontotermitis* sp. nov. isolated from the gut of termites

Kota Abe[1], Masahiro Yuki[2], Yumiko Imagawa[1], Atsushi Hisatomi[2], Moriya Ohkuma[2], Mitsuo Sakamoto[2,3,*] and Satoko Noda[1,*]

Author affiliations:

[1]Graduate School of Science and Engineering, Ibaraki University, Mito, Ibaraki 310-8512, Japan; [2]Microbe Division/Japan Collection of Microorganisms, RIKEN BioResource Research Center, Tsukuba, Ibaraki 305-0074, Japan; [3]NODAI Culture Collection Center, Tokyo NODAI Research Institute, Tokyo University of Agriculture, Setagaya-ku, Tokyo 156-8502, Japan.

***Correspondence:**

Satoko Noda, satoko.noda.z17@vc.ibaraki.ac.jp

Mitsuo Sakamoto, sakamoto@riken.jp

Table S1. *In silico* prediction of the DNA-DNA hybridization values, average nucleotide identities, average amino acid identities, and 16S rRNA gene sequence similarities between strain RyT2^T and reference strains.

Strain	16S rRNA (%)	GGDC formula 2 (%)	ANI (%)	AAI (%)
L. chungangensis DSM 22330^T	97.15	32.70	86.27	89.59
L. laudensis DSM 28961^T	96.71	36.70	88.01	90.49
L. reticulitermitis JCM 32106^T	96.13	31.70	86.03	89.36

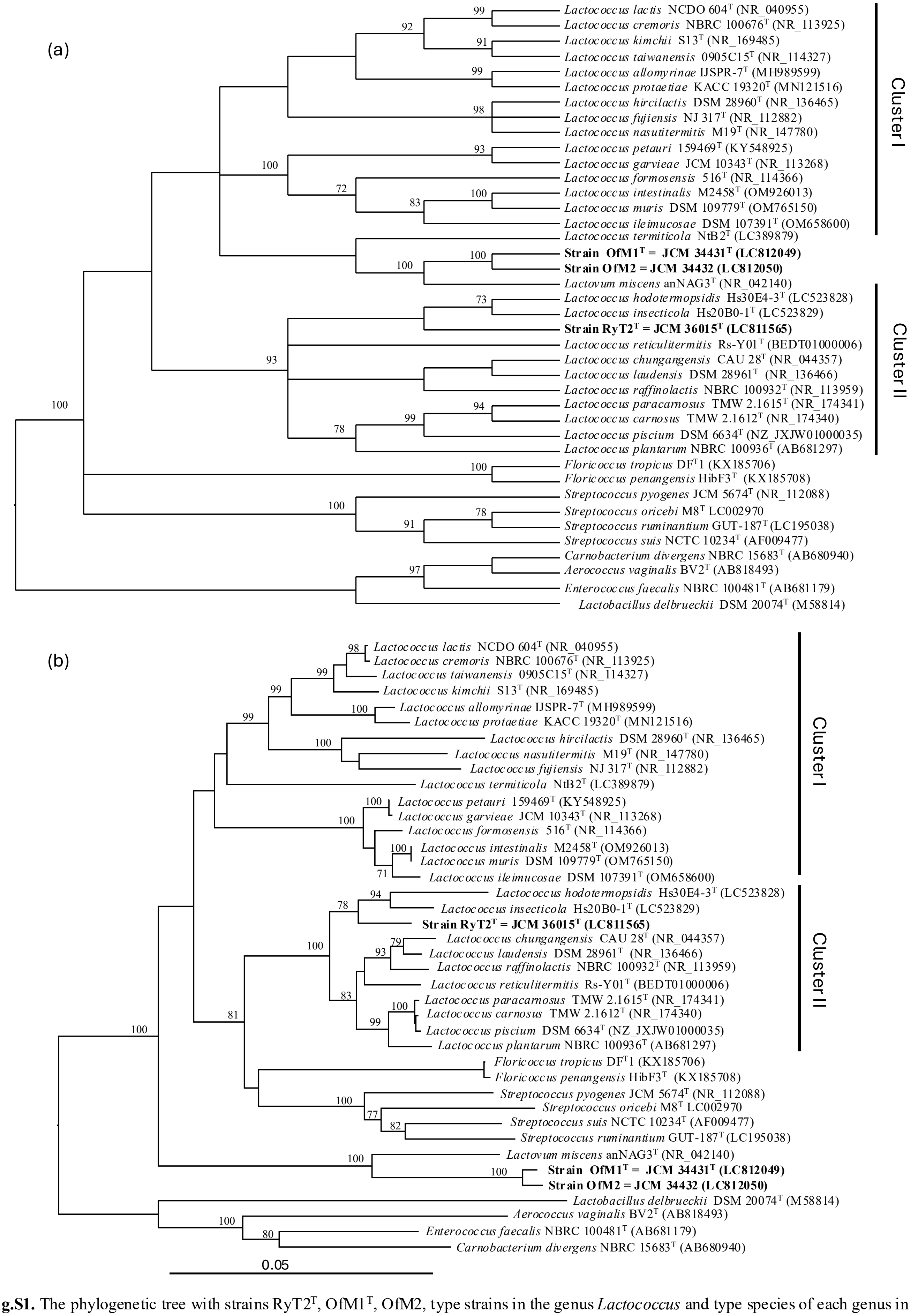

Fig.S1. The phylogenetic tree with strains RyT2^T, OfM1^T, OfM2, type strains in the genus *Lactococcus* and type species of each genus in the family *Streptococcaceae*, based on 16S rRNA gene sequences. (a) MP tree (b) NJ tree. The tree was inferred with unambiguously aligned nucleotide sites of 1343 for 16S rRNA. Bootstrap values are expressed as percentage of 1000 replications for MP and NJ analysis indicated at nodes. Values below 70% are not indicated. The outgroup taxa in the analyses were four species in *Lactobacillales* (*Lactobacillus delbrueckii, Aerococcus vaginalis, Carnobacterium divergens* and *Enterococcus faecalis*). The accession numbers of 16S rRNA genes are given in parentheses. Bar, 0.05 substitutions per nucleotide position.

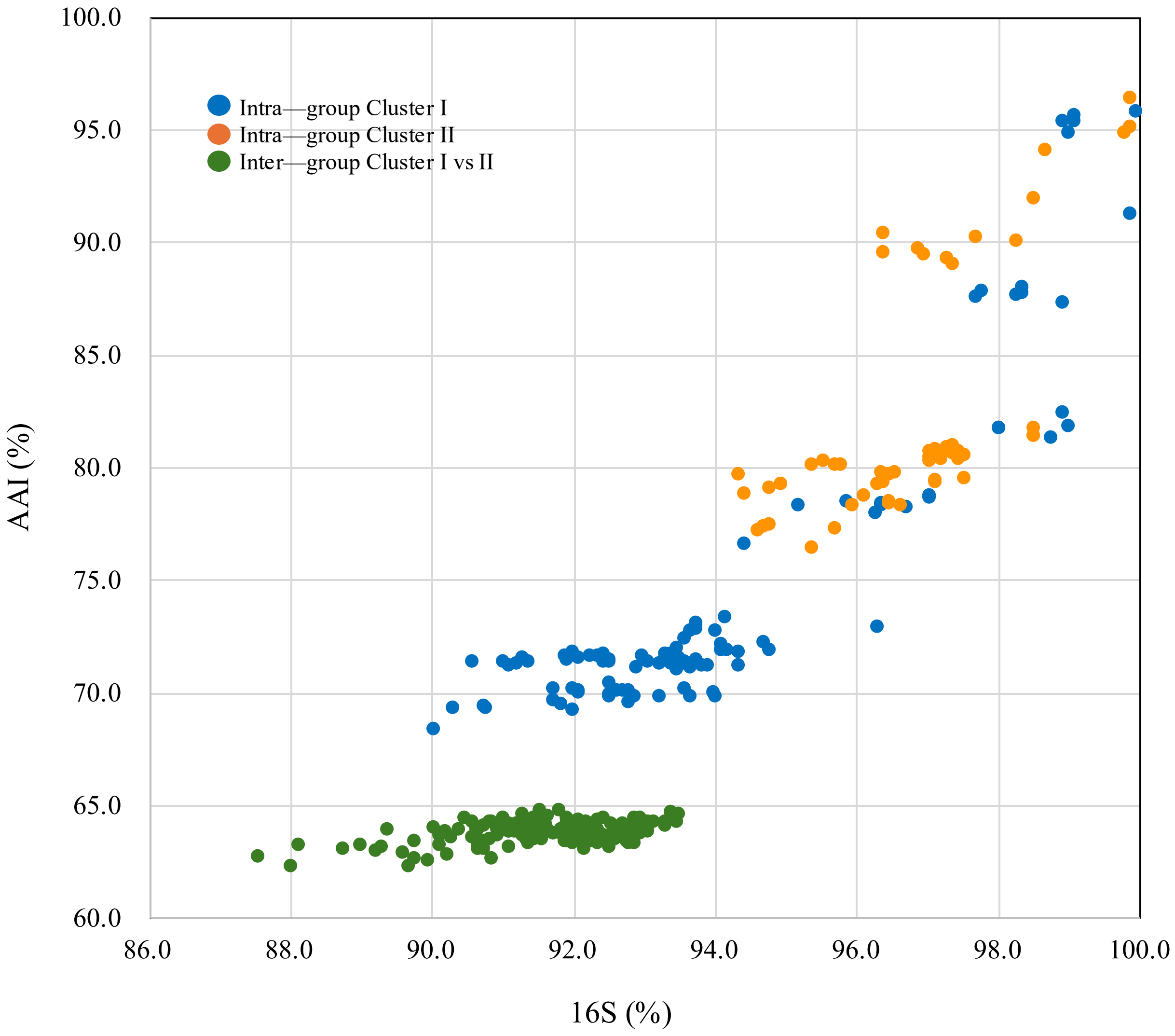

Fig. S2. Pairwise AAI in the genus *Lactococcus*. Genome sequences for type strains of all species in the genus *Lactococcus* were used.
